# Maturation of T and B Lymphocytes in the Assessment of the Immune Status in COVID-19 Patients

**DOI:** 10.3390/cells9122615

**Published:** 2020-12-05

**Authors:** Iwona Kwiecień, Elżbieta Rutkowska, Krzysztof Kłos, Ewa Więsik-Szewczyk, Karina Jahnz-Różyk, Piotr Rzepecki, Andrzej Chciałowski

**Affiliations:** 1 Laboratory of Hematology and Flow Cytometry, Department of Internal Medicine and Hematology, Military Institute of Medicine, Szaserów 128, 04-141 Warsaw, Poland; erutkowska@wim.mil.pl; 2 Department of Infectious Diseases and Allergology, Military Institute of Medicine, Szaserów 128, 04-141 Warsaw, Poland; kklos@wim.mil.pl (K.K.); achcialowski@wim.mil.pl (A.C.); 3 Department of Internal Medicine, Pulmonology, Allergology and Clinical Immunology, Military Institute of Medicine, Szaserów 128, 04-141 Warsaw, Poland; ewiesik-szewczyk@wim.mil.pl (E.W.-S.); kjrozyk@wim.mil.pl (K.J.-R.); 4 Department of Internal Medicine and Hematology, Military Institute of Medicine, Szaserów 128, 04-141 Warsaw, Poland; przepecki@wim.mil.pl

**Keywords:** COVID-19, flow cytometry, B cells maturation, T cells maturation, plasmablasts, CD4+ cells, effector CD8+ cells, central memory CD4+

## Abstract

Cell response to novel coronavirus disease 19 (COVID-19) is currently a widely researched topic. The assessment of leukocytes population and the maturation of both B and T lymphocytes may be important in characterizing the immunological profile of COVID-19 patients. The aim of the present study was to evaluate maturation of B and T cells in COVID-19 patients with interstitial lesions on chest X-ray (COVID-19 X-ray (+)), without changes on X-ray (COVID-19 X-ray (−)) and in healthy control. The study group consisted of 23 patients divided on two groups: COVID-19 X-ray (+) *n* = 14 and COVID-19 X-ray (−) *n* = 9 and control *n* = 20. The flow cytometry method was performed. We observed a significantly higher percentage of plasmablasts and lower CD4+ lymphocytes in COVID-19 X-ray (+) patients than in COVID-19 X-ray (−) and control. In the COVID-19 X-ray (+) patients, there was a lower proportion of effector CD4+ T cells, naïve CD8+ T cells and higher central memory CD4+ cells and effector CD8+ T cells than control. The above results showed that the assessment of selected cells of B and T lymphocytes by flow cytometry can distinguish patients with COVID-19 and differentiate patients with and without changes on chest X-ray.

## 1. Introduction

The novel coronavirus disease 19 (COVID-19) is caused by a coronavirus, called severe acute respiratory syndrome coronavirus-2 (SARS-CoV-2) a new strain not previously identified in humans [1,2]. The infection is usually associated with mild symptoms, ranging from low-grade fever, dry cough to fatigue and diarrhea, but may be responsible for severe interstitial pneumonia, myocarditis, acute kidney injury, acute respiratory distress syndrome (ARDS), multiorgan failure and death. In more severe conditions with dyspnea and respiratory impairment admission to intensive care unit (ICU) and advanced respiratory assistance are required. The lung represents the most commonly affected organ. Chest imaging can provide rapid and valuable information in the diagnosis of COVID-19 pneumonia [3]. As suggested in the recently published World Health Organization (WHO) advice guide for the diagnosis and management of COVID-19, chest imaging should be used for diagnostic purpose in symptomatic patients if real time polymerase chain reaction (RT-PCR) is not available or its results are delayed, or in case of negative result in the presence of high clinical suspicion of COVID-19 [4]. Chest X-rays are a useful diagnostic tool for assessing various lung diseases, such as pneumonia, but interpretation of the images can be challenging and time consuming. Clinicians need to have other methods which may indicate dysregulation of the immune system in COVID-19 infection. The pathophysiology of such virus is not yet completely understood [5]. Ongoing research have shown that pulmonary inflammation in COVID-19 patients is associated with increased plasma levels of proinflammatory and anti-inflammatory cytokines so-called “cytokine storm” [6,7,8]. Lymphocytes and the subsets: CD4+ T cells, CD8+ T cells, B cells, and natural killer (NK) cells play an important role in the maintenance of immune system function. After virus infection, alteration in total lymphocyte numbers and the subsets varies with different virus types, indicating a potential association between lymphocyte subset alteration and viral pathogenic mechanisms [9]. COVID-19 patients have a reduction in absolute numbers of lymphocytes, including CD4+ and CD8+ T lymphocytes, which display markers related to activation or exhaustion/senescence, along with altered expression of master regulators and several chemokine receptors [10,11,12]. Some research has suggested that the decreased number of mononuclear cells in the blood may be due to their migration directly to the infected lungs [13]. The differences in the percentage or absolute number of basic leukocyte subpopulations may be related to the severity of the disease and manifest themselves only in severe forms of COVID-19 infection [14,15].

Recent studies indicated a clear decrease in peripheral lymphocytes in COVID-19 patients but specific alteration in the subsets is still unknown. There are large gaps in understanding if T and B cell response profiling can be used as a prognostic biomarker of disease outcome in COVID-19 patients. It is known that B cells participate in the antiviral immune response. The generation of functioning B cells is preceded by the exit of the transitional B lymphocytes from the bone marrow. They migrate to the peripheral lymphoid organs and eventually develop into naive B cells [16]. Once exposed to an antigen, the naive B cells either become memory B cells (classical class-switched memory B cells and natural non-switched subsets) or plasmablasts that secrete antibodies upon T cell-mediated [17].

T cells development, of both CD4+ and CD8+, takes place in the thymus, from where recent thymic emigrants cells (RTE) exit and home to secondary lymphoid organs (spleen and lymph nodes). Maturation of RTE results in the generation of mature naive T cells which after contact with the antigen, transform into effector and central memory cells [18,19]. Effector T lymphocytes eliminate virus-infected cells while the central memory cells are activated after another antigens contact and become effector memory or central memory cells [20]. Mature CD4+ cells mainly play a role in the activation, proliferation and differentiation of CD8+ cytotoxic T lymphocytes and stimulation B lymphocytes to specific antibody formation. The main task of the T CD8+ cells is the elimination of viruses infected cells [21]. Moreover, little is known about these cells’ subpopulation contribution in specific immune response of patients with COVID-19.

In this study, we aimed to characterize peripheral lymphocyte subsets alteration mainly maturation B and T cells in COVID-19, which might help elucidate the pathogenesis and develop novel biomarkers and therapeutic strategies for COVID-19 infection.

## 2. Materials and Methods

### 2.1. Study Participants

Twenty three patients with SARS-CoV-2 were defined as positive from RT-PCR assay from nasal and pharyngeal swab specimens according to the WHO guidelines. Patients COVID-19+ were recruited from 10 May to 30 September 2020 at Military Institute of Medicine, at the Department of Infectious Diseases and Allergology.

There were 8 women and 15 men; mean age: 55.3 ± 19.1 years. Based on radiological interstitial lesions on chest X-ray patients were classified into two groups. Group one included 9 patients without any changes on chest X-ray (COVID-19 X-ray (−)), with stable lung parameters, with no oxygen supplementation or ventilation. Second group were 14 patients with interstitial densities in the lungs (COVID-19 X-ray (+)) without signs of pulmonary congestion, no fluid in the pleural cavities. Seven of them required oxygen ventilation and 3 required invasive ventilation (Table 1). The twenty age-matched healthy individuals were used as control group: 15 female, 5 male mean ages: 54.9 ± 10.1 years. Clinical characteristics of all COVID-19 patients were presented in Table 1.

Peripheral blood (PB) samples were collected from all COVID-19 patients 24–48 h after first examination and before administration of any antiviral and/or immunosuppressive drug, and from healthy individuals. The routine test of white blood cells count (WBC) was performed using a hematological analyzer Sysmex XN-1500 (Sysmex Corp., Kobe, Japan). The study was performed in accordance with Military Institute of Medicine Ethics Committee (number: 47/WIM/2020).

### 2.2. Flow Cytometry

Leukocytes and lymphocytes subset were performed by multiparameter flow cytometry method with panel of monoclonal antibodies using FACS Canto II BD flow cytometry (Becton Dickinson, Franklin Lakes, NJ, USA). For surface markers detection on leukocytes and lymphocytes T, B and NK subset cells were stained with fluorescently labelled antibodies: CD4-FITC (catalog number: 345768, clone number: SK3), CD56-PE (catalog number: 345810, clone number: MY31), CD3-PerCP-Cy5.5 (catalog number: 332771, clone number: SK7), CD19-PE-Cy7 (catalog number: 341113, clone number: SJ25C1), CD8-APC (catalog number: 345775, clone number: SK1), CD16-APC-H7 (catalog number: 560195, clone number: 3G8), HLA-DR-V450 (catalog number: 655874, clone number: L243) and CD45-V500 (catalog number: 655873, clone number: 2D1), (BD Bioscience).

To evaluate the lymphocyte B cells maturation were used following antibodies: CD19-FITC (catalog number: 363007, clone number: SJ25C1), IgD-PE (catalog number: 555779, clone number: -), CD27-PerCP-Cy5.5 (catalog number: 656643, clone number: L128), IgM-APC (catalog number: 551062, clone number: -), CD38-APCH7 (catalog number: 656646, clone number: HB7) and CD45-V500 (catalog number: 655873, clone number: 2D1), (BD Bioscience). We distinguished the following subpopulations in B-cell maturation [20,22].

- Transitional B cells: IgM++ IgD++ CD38++ CD27- CD19+ CD45+
- Naïve B cells: IgM+ IgD++ CD38+ CD27- CD19+ CD45+
- Non-switched memory B cells (marginal zone-like B cells): IgM++ IgD+ CD38+ CD27+ CD19+ CD45+
- Class switched memory B cells: IgM- IgD- CD38+ CD27+ CD19+ CD45+
- Plasmablasts: IgM-/+ IgD- CD38+++ CD27++ CD19+ CD45+

To evaluate the lymphocyte T CD4+ and CD8+ maturation were used following antibodies: CD4-FITC (catalog number: 345768, clone number: SK3), CD8-V450 (catalog number: 560347, clone number: RPA-T8), CD196-PE (catalog number: 551773, clone number: -), CD197-PerCP-Cy5.5 (catalog number: 353220, clone number: G043H7 BioLegend (San Diego, CA, United States), CD45RO-PE-Cy7 (catalog number: 560608, clone number: UCHL1), CD45RA-APC (catalog number: 550855, clone number: -), CD62L-PE (catalog number: 555544, clone number: -) and CD31-PerCP-Cy5.5 (catalog number: 566563, clone number: WM59) (BD Biosciences). We distinguished the following subpopulations in T-cell CD4+ or CD8+ maturation [20].

- Recent thymic emigrants T cells: CD45RA+ CD62L+ CD31+ CD3+ CD45+
- Naïve T cells: CD45RA+ CD197+ CD3+ CD45+
- Effector T cells: CD45RA+ CD197- CD3+ CD45+
- Central memory T cells: CD45RO+ CD197+ CD3+ CD45+
- Effector memory T cells: CD45RO+ CD197- CD3+ CD45+

**Figure 1:**
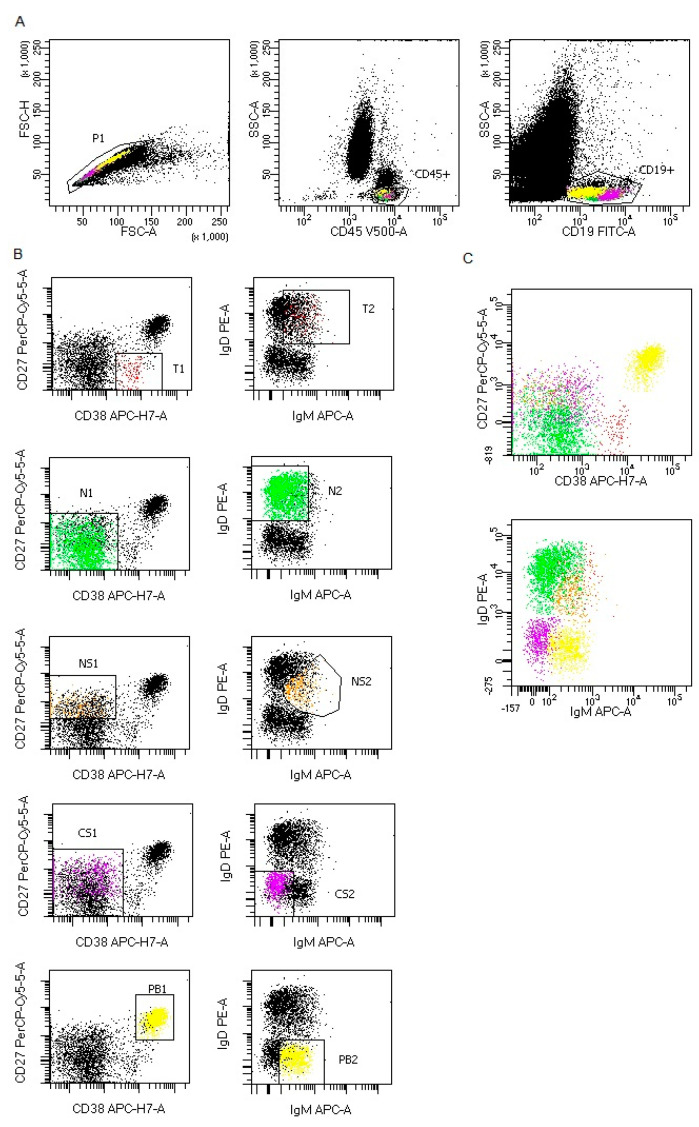
Representative B lymphocytes maturation gating strategy in COVID-19 patients. (**A**) B lymphocytes gating strategy: FSC-A vs. FSC-H plot: Gating the cells that have an equal area and height, thus removing clumps (greater FSC-A relative to FSC-H) and debris (very low FSC), CD45 vs. SSC-A plot: Broad selection of lymphocytes based on their SSC/CD45 properties, CD19 vs. SSC-A plot: Broad selection of lymphocytes B based on their SSC/CD19 properties. (**B**) B lymphocytes maturation gating strategy for each maturation subsets: T1,T2-transitional B cells, N1,N2-naïve B cells, NS1, NS2-non-switched memory B cells, CS1, CS2-class switched memory B cells and PB1, PB2-plasmablasts. (**C**) representative plots with all B lymphocytes maturation subsets. CD27 vs. CD38 plot: Broad selection of B lymphocyte maturation subsets based on their CD27/CD38 properties, IgD vs. IgM plot: Broad selection of B lymphocyte maturation subsets based on their IgD/IgM properties. Plots show all B lymphocyte maturation subsets: Transitional B cells (red), naïve B cells (green), non-switched memory B cells (orange), class switched memory B cells (purple) and plasmablasts (yellow).

**Figure 2:**
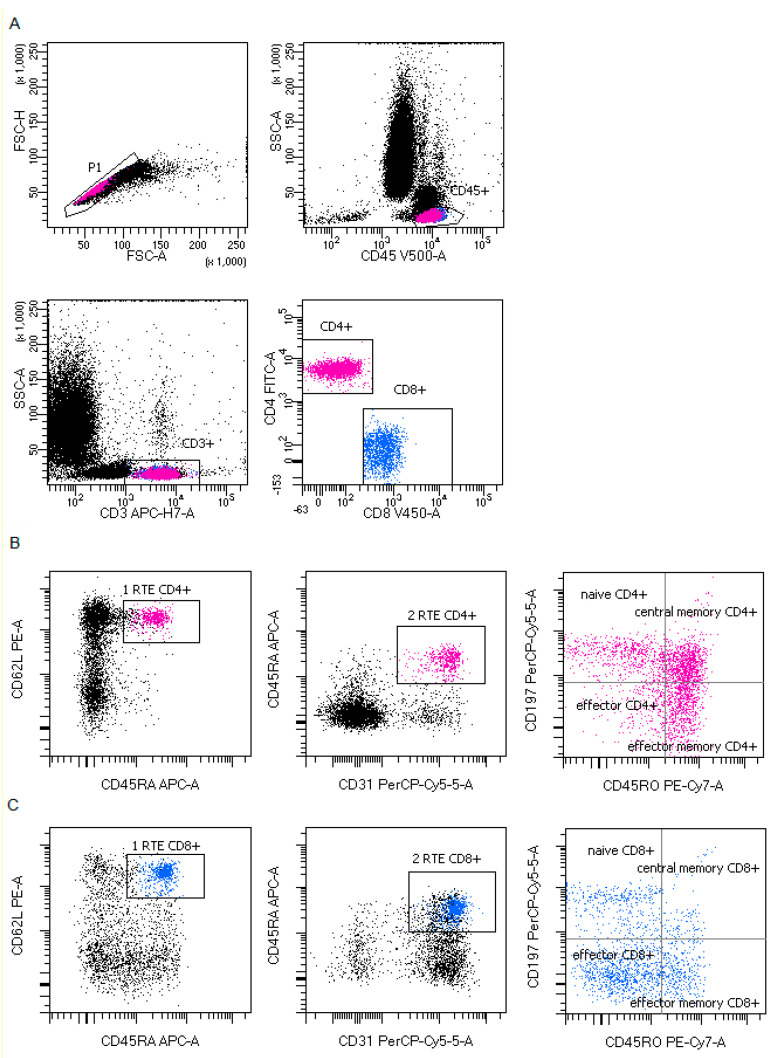
Representative T lymphocytes maturation gating strategy in coronavirus disease 19 (COVID-19) patients. (**A**) T lymphocytes gating strategy: FSC-A vs. FSC-H plot: Gating the cells that have an equal area and height, thus removing clumps (greater FSC-A relative to FSC-H) and debris (very low FSC), CD45 vs. SSC-A plot: Broad selection of lymphocytes based on their SSC/CD45 properties, CD3 vs. SSC-A plot: Broad selection of lymphocytes T based on their SSC/CD3 properties. CD4 vs. CD8 plot: Broad selection of lymphocytes T CD4+ (pink) and CD8+ (blue) based on their CD4/CD8 properties. (**B**) CD4+ T lymphocytes maturation gating strategy for each maturation subsets. CD62L vs. CD45RA plot and CD45RA vs. CD31: Broad selection of CD4+ T lymphocytes based on their CD62L/CD45RA and CD45RA vs. CD31 properties and allow select recent thymic emigrants T CD4+ cells RTE (plot 1: RTE CD4+ and plot 2: RTE CD4+), CD197 vs. CD45RO plot: Broad selection of CD4+ T lymphocytes based on their CD197/CD45RO properties: naïve CD4+ T cells, effector CD4+ T cells, central memory CD4+ T cells and effector memory CD4+ T cells. (**C**) CD8+ T lymphocytes maturation gating strategy for each maturation subsets. CD62L vs. CD45RA plot and CD45RA vs. CD31: Broad selection of CD8+ T lymphocytes based on their CD62L/CD45RA and CD45RA vs. CD31 properties and allow selected recent thymic emigrants T CD8+ cells RTE (plot 1: RTE CD8+ and plot 2: RTE CD8+), CD197 vs. CD45RO plot: Broad selection of CD8+ T lymphocytes based on their CD197/CD45RO properties: Naïve CD8+ T cells, effector CD8+ T cells, central memory CD8+ T cells and effector memory CD8+ T cells.

Representative B and T lymphocytes maturation gating strategy in COVID-19 patients were presented in Figure 1; Figure 2.

Samples were incubated for 20 min in room temperature. After two washing, cells were analyzed within 2 h. For each sample, a minimum of 20,000 events were collected. Data were analyzed with DIVA Analysis software 8.0.1 (BD) and Infinicyt 1.8 Flow Cytometry (Cytognos, Salamanca, Spain).

### 2.3. Statistical Analysis

All statistical analyses were performed using the Statistica 13.0 software (TIBCO Software, Palo Alto, CA, USA). A *p* < 0.05 was considered as statistically significant. The results are expressed as means and SDs, medians with interquartile range (Q1–Q3). For group comparison the Kruskal–Wallis with the post-hoc Wilcoxon’s signed rank test were used. Differences were considered statistically significant when *p* < 0.05. For graphic processing was used Prism GraphPad (Version 7, GraphPad Software, La Jolla, CA, USA).

## 3. Results

To assess the maturation of B and T lymphocytes after SARS-CoV-2 infection, we analyzed the blood of 23 COVID-19 patients using multi-parameter flow cytometry. The clinical characteristics of the investigated group were summarized in Table 1. COVID-19 patients were divided into two groups with interstitial lesions on chest X-ray (*n* = 14 COVID-19 X-ray (+)) and without changes on X-ray (*n* = 9 COVID-19 X-ray (−)) and they were compared to healthy group (*n* = 20). Median proportions of absolute number of basic leukocytes and lymphocytes subtypes were presented in Table 2 and the percentages of basic leukocytes and lymphocytes subtypes were shown in in the Appendix A, Table A1. In the COVID-19 X-ray (+) we noticed a significantly lower median absolute number of lymphocytes, lymphocytes T including CD4+ T lymphocytes and lymphocytes B than in healthy group. We did not observed differences in count of lymphocytes between patients with changes on X-ray and without changes on X-ray. Basophils and eosinophils absolute number were also significantly lower in COVID-19 X-ray (+) patients than in heathy donors. Taking into account the percentages of the analyzed basic leukocyte subpopulations, similar statistical significances were observed for: T lymphocytes, CD4 cells, eosinophils as in the case of the absolute numbers analysis between COVID-19 X-ray (+) patients and heathy donors (Table 2). Additionally, the difference in CD4+ cells median proportion between COVID-19 X-ray (−) and COVID-19 X-ray (+) was observed (respectively, 23.1% vs. 8.0%, *p* = 0.0026). The median percentage of neutrophils was significantly higher in COVID-19 X-ray (+) then in COVID-19 X-ray (−) patients (respectively, 62.9% vs. 37.6%, *p* = 0.0229).

### 3.1. B Cells Maturation

**Figure 3:**
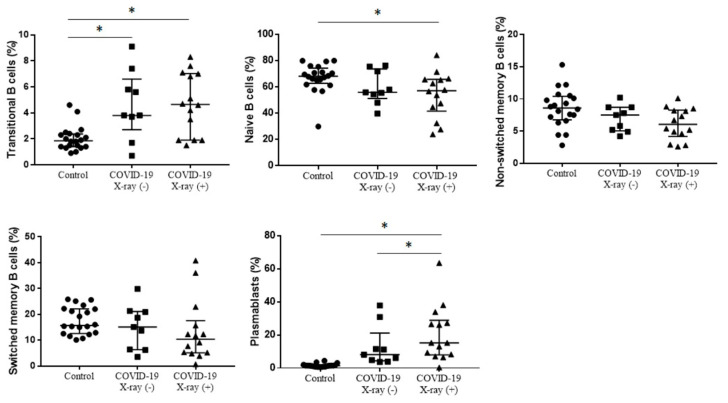
The differences in the median proportion of B lymphocytes maturation subsets: Transitional B cells, naïve B cells, non-switched memory B cells, class switched memory B cells and plasmablasts between healthy patients (Control), patients with COVID-19 without interstitial lesions on chest X-ray (COVID-19 X-ray (−)) and with interstitial lesions on chest X-ray (COVID-19 X-ray (+)). Graphs show the median values and quartile Q1—Q3 (* *p* < 0.05).

There was higher median proportion of transitional B cells in COVID-19 X-ray (+) patients and COVID-19 X-ray (−) patients than in healthy control (respectively, 4.6% vs. 3.8% vs. 1.8%, *p* = 0.0016), without differences between COVID-19 positive patients with interstitial lesions on chest X-ray and no interstitial lesions on chest X-ray. In this study we observed significantly lower median proportion of naïve B cells in COVID-19 X-ray (+) patients than in healthy control, without differences between COVID-19 X-ray (+) patients and COVID-19 X-ray (−) patients (57.0% vs. 55.8% vs. 68.0%, *p* = 0.0279). When we analyzed the median proportion of plasmablasts we noticed the highest proportion in COVID-19 X-ray (+) patients and, respectively, significantly higher in COVID-19 X-ray (+) patients than in COVID-19 X-ray (−) patients and in healthy control (15.2% vs. 8.1% vs. 1.4%, *p* < 0.0001). There were not differences in proportion of non-switched memory B cells and switched memory B cells between COVID-19 X-ray (+) patients, COVID-19 X-ray (−) patients and healthy control (Figure 3, Table 3).

**Figure A1:**
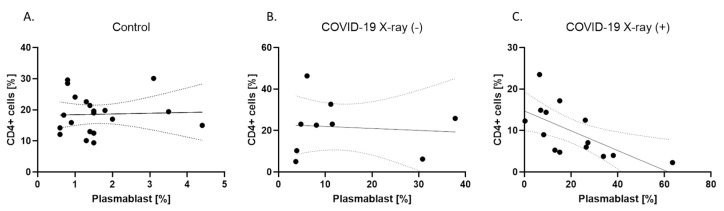
The correlations between proportion of plasmablasts and proportion of CD4+ cells in healthy patients (**A**), patients with COVID-19 without interstitial lesions on chest X-ray ((**B**): COVID-19 X-ray (−)) and patients with COVID-19 with interstitial lesions on chest X-ray ((**C**): COVID-19 X-ray (+)).

When we analyzed correlation in each group we observed a significantly negative correlation between plasmablasts and CD4+ cells in COVID-19 X-ray (+) patients (R = −0.73, *p* < 0.05), without this correlation in COVID-19 X-ray (−) patients and healthy control. The correlation analyses were presented in the Appendix A, Figure A1.

### 3.2. T Cells Maturation

**Figure 4:**
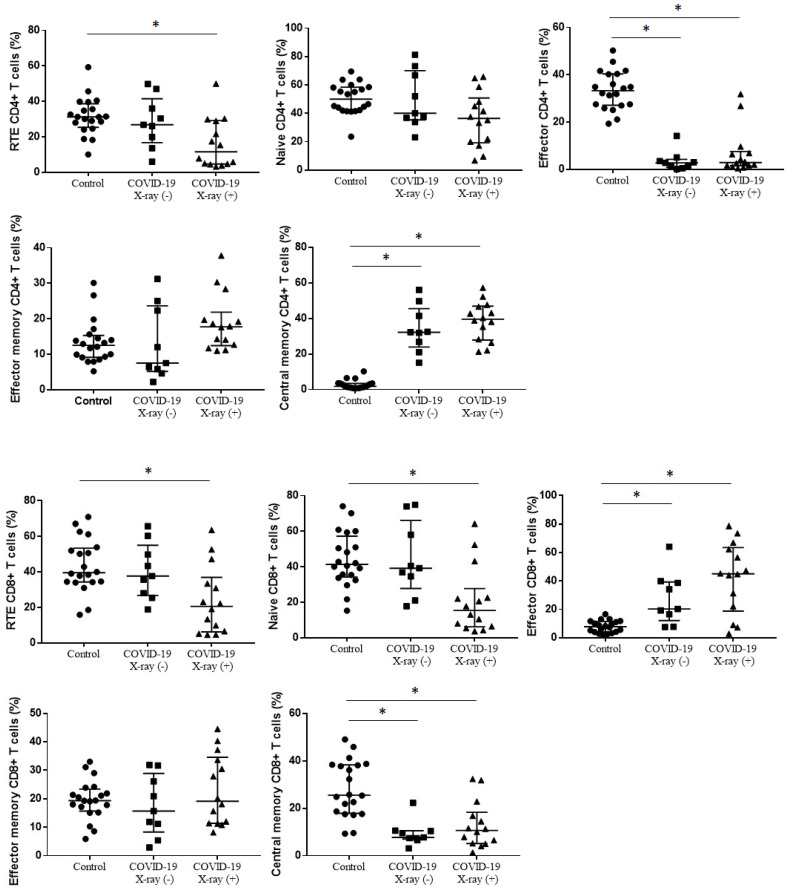
The differences in the median proportion of T lymphocytes maturation (CD4+ and CD8+) subsets: Recent thymic emigrants T cells (RTE), naïve T cells, effector T cells, central memory T cells and effector memory T cells between healthy patients (Control), patients with COVID-19 without interstitial lesions on chest X-ray (COVID-19 X-ray (−)) and with interstitial lesions on chest X-ray (COVID-19 X-ray (+)). Graphs show the median values and quartile Q1–Q3 (* *p* < 0.05).

Taking into account the differences between the groups in the maturation of T cells (Figure 4, Table 3) the median proportion of RTE CD4+ T cells was significantly lower in COVID-19 X-ray (+) patients than in healthy control, without differences between COVID-19 X-ray (+) patients and COVID-19 X-ray (−) patients (11.6% vs. 26.8% vs. 31.2%, *p* = 0.0052). The median proportions of effector CD4+ T cells were significantly lower in COVID-19 X-ray (+) and COVID-19 X-ray (−) patients than in healthy control (2.9% vs. 2.8% vs. 33.2%, *p* < 0.0001). We observed higher median proportion of central memory CD4+ T cells in COVID-19 X-ray (+) patients than in CD4+ T cells in COVID-19 X-ray (−) and significantly the lowest in healthy control (respectively, 39.5% vs. 32.2% vs. 1.8%, *p* < 0.0001). The median proportions of effector memory CD4+ T cells and naïve CD4+ T cells did not differ between COVID-19 X-ray (+) patients, COVID-19 X-ray (−) patients and healthy control.

For CD8+ T cells maturation the median proportion of RTE CD8+ T cells was significantly lower in COVID-19 X-ray (+) patients than in healthy control, without differences between COVID-19 X-ray (+) patients and COVID-19 X-ray (−) patients (20.6% vs. 37.7% vs. 39.5%, *p* = 0.0052). We observed lower median proportion of naïve CD8+ T cells in COVID-19 X-ray (+) patients than in COVID-19 X-ray (−) patients and healthy control, without significant differences between COVID-19 X-ray (+) patients and COVID-19 X-ray (−) patients (15.4% vs. 39.2% vs. 42.4%, *p* = 0.0029). The median proportion of central memory CD8+ T cells was lower in COVID-19 X-ray (+) patients than in healthy control and also lower in COVID-19 X-ray (−) patients than in healthy control (respectively, 10.7% vs. 25.5% and 7.7% vs. 25.5%, *p* = 0.0001). We observed higher median proportion of effector CD8+ T cells in COVID-19 X-ray (+) patients than in COVID-19 X-ray (−) patients and healthy control, without significant differences between COVID-19 X-ray (+) patients and COVID-19 X-ray (−) patients (45.0 vs. 20.3 vs. 7.8%, *p* = 0.0001). The median proportions of effector memory CD8+ T cells did not differ between COVID-19 X-ray (+) patients, COVID-19 X-ray (−) patients and healthy control (Figure 4, Table 3).

## 4. Discussion

### 4.1. Leukocytes and Lymphocytes Subsets in COVID-19 Patients

In this study, we focused on characterizing the immunological profile of COVID-19 patients by examining subpopulations of T and B lymphocyte maturation. Most of the patients exhibited typical clinical manifestations for COVID-19 infection like: Fever, cough, dyspnea and fatigue. Acute respiratory failure requiring mechanical ventilation was reported in three patients (Table 1). Depending on the presence or absence of radiographic interstitial lesions on the chest X-ray, patients were divided into two groups to distinguish individuals with different degrees of disease severity. We found a lower proportion of T lymphocytes (CD4+ and CD8+ subsets), B lymphocytes, eosinophils and basophils in COVID-19 X-ray (+) patients than in healthy controls but we did not observe the differences in absolute number of analyzed leukocytes subpopulations between patients with and without lung lesions on chest X-ray. The results of other researchers confirmed our observations [10,14]. Moreover, they have shown that decreased of absolute number of leukocytes may be associated with the severity of the disease. Moratto, D. et al. [14] presented that flow cytometry analysis revealed significant differences in number of lymphocytes among patients with moderate disease, severe and a critical phenotype. The absolute counts of CD3+, CD4+ and CD8+ lymphocytes were different in those three groups of patients. Liu, Z. et al. [23] found that low counts of CD4+ and CD8+ T lymphocytes were more common in patients with severe type of COVID-19 infection. Odak, I. et al. [15] also have shown that absolute numbers of lymphocyte subsets were significantly decreased in COVID-19 patients according to clinical severity. In severe type of disease all lymphocyte analyzed subsets were reduced, whilst in mild disease were at the level of healthy control. Sun H.B. et al. [24] have shown that the lymphopenia in patients with COVID-19 was mainly manifested by decreases in the CD4+ T lymphocyte number and correlated with the severity of COVID-19 disease. Zhang W. et al. [25] have highlighted that lymphocyte subsets are the indicators of severity of COVID-19 disease. In our study we observed significantly lower median proportion of CD4+ cells and higher median proportion of neutrophils in COVID-19 X-ray (+) than in COVID-19 X-ray (−). The presence of neutrophilia in COVID-19 patients has been also reported by other researchers. Henry B. et al. [26] have found a significant association of neutrophilia with progression to severe course of the disease. Qun S. et al. [27] have shown that high neutrophil-to-lymphocyte ratio was closely associated with the severity of non-mild COVID-19 infection. In contrast, some studies in COVID-19 patients showed neutrophils within the normal range [7,28,29], which was also found in our study.

In our results we observed a significant difference between COVID-19 X-ray (+) and COVID-19 X-ray (−) patients in percentages of analyzed leukocytes subpopulation, not in absolute numbers. This finding can be attributed to the fact that most of our COVID-19 patients (with and without chest X-ray changes) were moderately infected.

The above discussed results confirmed that in patients with severe COVID-19 stage, finding significant changes in the elements of the immune system could be easier. Therefore, it seems reasonable to accurately characterize the B and T lymphocyte subpopulations at different stages of maturation in patients with mild to moderate type of COVID-19 infection.

### 4.2. B Cells Maturation in COVID-19 Patients: Role of Plasmablast and Transitional Cells

We have observed that COVID-19 X-ray (+) patients had the highest percentage of plasmablasts and increased percentage of transitional B lymphocytes. Moreover, we observed a decreased number of total B cells and naïve B cells in COVID-19 X-ray (+) patients when we compared to healthy control. Some researchers also revealed massive plasmablasts infiltration in COVID-19 patients [30]. De Biasi, S. et al. [31] found a decreased number of total and naïve B cells, along with decreased numbers of memory switched and non-switched B cells with significant increase of plasmablasts. Otherwise, B lymphocytes showed a normal proliferation index and number of dividing cells per cycle. The alterations in the B-cell compartment may underline the immune system’s effort to make up for lymphopenia with the increase in transitional B cells and plasmablasts. Vaisman–Mentesh, A. et al. [32] hypothesized that the differentiation of B cells is impaired after infection with SARS-CoV-2. They also found that short-lived plasmablasts were a major contributor to the high levels of serum antibodies. Mathew D. et al. [30] have also presented that plasmablast responses were present in COVID-19 patients, reaching > 30% of total B cells.

Moreover, we observed a negative significant correlation between CD4+ cells and plasmablasts in COVID-19 X-ray (+) patients, which was not observed in the other study groups and has not been yet presented by other authors.

In summary, the decline in proportion of CD4+ cells, the infiltration of plasmablasts and the above-mentioned correlation, allowed to differentiate COVID-19 X-ray (+) patients and COVID-19 X-ray (−) patients and could be used as a predictive factor for the occurrence of changes in the lungs.

### 4.3. T Cells Maturation in COVID-19 Patients: Role of Central Memory CD4+ T Cells and Effector CD8+ T Cells

We noticed a significant reduction of CD4+ effector cells and increase of CD4+ central memory cells, both in COVID-19 X-ray (+) and COVID-19 X-ray (−) compared to healthy group. These results confirmed that these changes appeared in COVID-19 patients regardless of the presence of lung lesions and, therefore, the severity of the disease. It could be hypothesized that lymphopenia in COVID-19 patients was due to not only reduction of total CD4+ cells, but a significant decrease of CD4+ effector cells. A decrease of CD4+ effector cells in COVID-19 patients was also observed by other researchers, in patients with moderate and severe type of COVID-19 infection compared to healthy volunteers [15]. The reduction in the number of these cells could be explained by their recruitment to the lungs or other infected organs, or cells damage caused by massive release of inflammatory mediators in response to infection [7,13,30]

The significant increase of CD4+ central memory cells, both in COVID-19 X-ray (+) and COVID-19 X-ray (−), in relation to the control group, might indicate appearance of immune memory in patients with COVID-19 infection, regardless of the occurrence of changes in lungs. A similar observation has been shown by other authors. Peng, Y. et al. [33] have characterized T cells which presented a mixed effector and central memory phenotype. Odak, I. et al. [15] have also shown that central memory CD4+ cells were dominated among CD4+ T lymphocytes, while percentages of CD4+ effector cells were reduced. Gong F. et al. [34] also observed that SARS-CoV-2-specific CD4+ T cells in blood were typically of central memory phenotype. Sekine, T. et al. [35] have presented that in patients with acute-phase of COVID-19 infection specific T cells displayed a highly activated cytotoxic phenotype, whereas in convalescent-phase T cells were polyfunctional and displayed a memory phenotype, the same in asymptomatic individuals and mild patients. The fact that memory T cells have generated in response to COVID-19 infection suggested that mild infection may elicit protection from severe COVID-19 in the future. However, in our study, the percentage of CD8+ central memory cells was lower in the COVID-19 groups compared to controls. Among the CD8+ cells, the subpopulation of CD8+ effector cells in COVID-19 patients were dominant. Odak, I. et al. [15] similar to our observation have shown that patients in mild course had higher proportion of CD8+ effector T cells than healthy control. Gong F. et al. [34] have also shown that SARS-CoV-2-specific CD8+ T-cells typically had a more effector phenotype. Westmeier, J. et al. [36] found that SARS-CoV-2 infection induced a cytotoxic response of CD8+ T cells, characterized by the simultaneous production of granzyme A and B as well as perforin. Moreover, they indicated that CD8+ cells were not functionally exhausted.

We concluded that in patients with COVID-19 infection, regardless of the existence of lung lesions, in both the mild and moderate COVID-19 groups, the organism is directed to the effector profile of the CD8+ cell population, and the memory profile of the CD4+ cells.

In addition, in our study we observed significantly lower proportions of both CD4+ and CD8+ RTE cells and naïve cells in COVID-19 X-ray (+) patients compared to the group of healthy volunteers, without differences between COVID-19 X-ray (+) patients and COVID-19 X-ray (−) patients. There are no reports of involvement of RTE cells in COVID-19 infection course in the literature. It is known that RTE represent a source of antigen-naïve T cells that enter the periphery throughout life. Some studies confirmed that the fundamental processes that are required to generate new T cells are affected by viruses and how these permutations affect reconstitution are not well understood. It is observed that chronic virus infection caused CD8+ T cell-mediated thymic destruction and impaired negative selection of lymphocytes T [37].

Interestingly, COVID-19 patients with changes on chest X-ray and no changes did not differ from each other, taking into account the above-mentioned subpopulations of T lymphocytes in contrast to percentage of CD4+ cells and the plasmablasts in B cell subpopulation. On the other hand, we observed many differences in the maturation of T cells between the COVID-19 group with lung lesions and healthy controls.

## 5. Conclusions

In this study, we characterized peripheral cell subsets of T and B lymphocytes maturation and their differences between COVID-19 subjects with and without chest X-ray changes and control group. We proposed that effector T CD8+ cells and memory T CD4+ cell response together with plasmablasts provided a reliable measurement of immune status that may be useful for evaluating COVID-19 patients. Additionally, the reduced percentage of CD4+ lymphocytes and the increased proportion of plasmablasts allowed to distinguish patients with changes in the lungs, which can set the direction for further research and constitute an additional element of diagnostics.

## Figures and Tables

**Table 1:** Patients’ characteristics.

ID	Age	F/M	Symptoms	PEMC	Chest X-ray Changes	Oxygen Suplementation	Invasive Ventilation	C
1	80	m	Fever, dyspnea, diarrhea, fatigue	yes	yes	no	no	yes
2	78	f	Fever, cough, dyspnea, fatigue	yes	yes	yes	no	yes
3	69	m	Fever, fatigue	yes	yes	yes	yes	yes
4	63	m	Fever	yes	yes	yes	no	yes
5	42	m	Fatigue	no	no	no	no	yes
6	39	f	Fatigue	no	no	no	no	yes
7	44	m	Fever, cough, dyspnea	yes	yes	no	no	yes
8	37	f	Fever, cough, dyspnea, diarrhea	yes	yes	no	no	yes
9	74	m	Fever, cough, dyspnea	yes	yes	yes	yes	yes
10	35	f	Fever, cough, dyspnea, fatigue	no	no	no	no	yes
11	57	m	Fever, cough, dyspnea	no	yes	no	no	yes
12	78	f	Fever, cough	yes	no	no	no	yes
13	39	f	Fatigue	yes	no	no	no	yes
14	72	m	Fever, cough, dyspnea, diarrhea, fatigue	yes	yes	yes	no	yes
15	28	m	Fever, cough, dyspnea, fatigue	no	yes	no	no	yes
16	63	m	Fever, cough	yes	yes	no	no	yes
17	43	m	Fatigue	yes	no	no	no	yes
18	33	m	Fever, cough, diarrhea, fatigue	no	no	no	no	yes
19	80	m	Fever, cough, dyspnea, fatigue	yes	yes	yes	yes	no
20	67	f	Fever, cough, dyspnea, fatigue	no	yes	yes	no	yes
21	72	m	Fever, cough, dyspnea, diarrhea, fatigue	no	yes	no	no	yes
22	47	f	Fever, cough, dyspnea, diarrhea, fatigue	no	no	no	no	yes
23	34	m	Fever, cough, dyspnea, diarrhea, fatigue	no	no	no	no	yes

Abbreviations: C: Convalescent, f: Female; m: Male; and PEMC: Pre-existing medical conditions.

**Table 2:** Differences in the median of white blood cells (WBC) count and leukocytes and main lymphocytes subpopulation counts between healthy patients (**A**), patients with COVID-19 without interstitial lesions on chest X-ray ((**B**): COVID-19 X-ray (−)) and patients with COVID-19 with interstitial lesions on chest X-ray ((**C**): COVID-19 X-ray (+)). Data expressed as median (Q1–Q3).

[k/µl]	Control Group n = 20 (A) Median (Q1–Q3)	COVID-19 X-ray (-) n = 9 (B) Median (Q1–Q3)	COVID-19 X-ray (+) n = 14 (C) Median (Q1–Q3)	*p* < 0.05 * Group A-B-C ANOVA, Kruskal-Wallis	*p* < 0.05 * Group, in Groups Post-Hoc
WBC	6 555 (4930–7535)	4580 (4150–6840)	4515 (3620–6810)	0.1954	-
Lymphocytes	2038 (1839–2934)	1350 (1087–2992)	961 (753–1799)	* 0.0080	* A-C 0.0056
T Lymphocytes	1677 (1384–2384)	951 (683–2253)	691 (524–1416)	* 0.0035	* A-C 0.0023
CD4 cells	978 (756–1560)	619 (533–1210)	319 (239–584)	* 0.0018	* A-C 0.0012
CD8 cells	625 (457–791)	313 (225–862)	330 (160–549)	0.0811	-
Ratio CD4/CD8	1.8 (1.5–2.3)	2.1 (1.4–3.6)	1.3 (0.6–2.4)	0.1582	-
B Lymphocytes	216 (190–284)	147 (115–186)	137 (77–238)	* 0.0056	* A-B 0.0234 *A-C 0.0245
NK cells	245 (204–447)	299 (170–445)	164 (101–397)	0.4313	-
Neutrophils	3310 (2139–4348)	1722 (1556–2880)	2896 (1611–4465)	0.0511	-
Eosinophils	160 (60–251)	69 (46–178)	45 (0–91)	* 0.0153	* A-C 0.0155
Basophils	30 (20–54)	27 (14–54)	6 (0–22)	* 0.0219	* A-C 0.0220
Monocytes	540 (381–690)	336 (280–397)	408 (246–669)	0.1340	-

**Table 3:** Differences in the proportion of B and T lymphocytes maturation between healthy patients (**A**), patients with COVID-19 without interstitial lesions on chest X-ray ((**B**): COVID-19 X-ray (−)) and patients with COVID-19 with interstitial lesions on chest X-ray ((**C**): COVID-19 X-ray (+)). Data expressed as median (Q1–Q3).

Cells Subsets: [% of B or T cells]	Control n = 20 (A) Median (Q1–Q3)	COVID-19 X-ray (−) n = 9 (B) Median (Q1–Q3)	COVID-19 X-ray (+) n = 14 (C) Median (Q1–Q3)	*p* < 0.05 * Group A-B-C ANOVA, Kruskal-Wallis	*p* < 0.05 * Group, in Groups Post-Hoc
B cells maturation:					
Transitional B	1.8 (1.4–2.3)	3.8 (3.7–5.8)	4.6 (1.9–7.0)	* 0.0016	* A-B 0.0334 * A-C 0.0031
Naïve B	68.0 (63.5–73.1)	55.8 (54.3–71.7)	57.0 (44.5–65.5)	* 0.0279	* A-C 0.0298
Non-switched memory	8.6 (6.9–10.3)	7.5 (5.2–8.7)	6.0 (4.6–8.2)	0.0605	-
Class switched memory	17.6 (12.7–22.8)	15.1 (6.4–20.9)	10.4 (5.2–15.7)	0.0627	-
Plasmablasts	1.4 (0.8–1.6)	8.1 (4.8–11.5)	15.2 (8.3–27.2)	* < 0.0001	* A-C < 0.0001 * B-C 0.0014
T cells maturation:					
Recent thymic emigrants (RTE) CD4	31.2 (26.3–37.6)	26.8 (19.9–36.0)	11.6 (4.8–29.0)	* 0.0052	* A-C 0.0040
Naïve CD4	50.0 (42.1–58.3)	40.1 (36.9–66.9)	36.4 (20.0–48.2)	0.0737	-
Effector CD4	33.2 (27.2–40.3)	2.8 (1.2–3.5)	2.9 (1.7–6.9)	* < 0.0001	* A-B < 0.0001 * A-C < 0.0001
Effector memory CD4	12.5 (9.2–15.0)	7.5 (5.8–22.3)	17.7 (12.7–19.7)	0.0503	-
Central memory CD4	1.8 (1.1–3.4)	32.2 (26.8–41.4)	39.5 (28.3–46.6)	* < 0.0001	* A-B 0.0002 * A-C < 0.0001
Recent thymic emigrants (RTE) CD8	39.5 (34.4–52.9)	37.7 (28.1–49.8)	20.6 (6.6–33.5)	* 0.0089	* A-C 0.0079
Naïve CD8	42.4 (35.5–59.7)	39.2 (34.6–58.1)	15.4 (6.5–22.6)	* 0.0029	* A-C 0.0027
Effector CD8	7.8 (4.1–11.4)	20.3 (16.5–38.6)	45.0 (22.1–62.4)	* 0.0001	* A-B 0.0206 * A-C 0.0002
Effector memory CD8	19.3 (16.2–22.9)	15.7 (11.2–26.1)	19.1 (11.4–33.7)	0.6454	-
Central memory CD8	25.5 (18.1–38.2)	7.7 (7.4–10.4)	10.7 (5.2–16.8)	* 0.0001	* A-B 0.0006 * A-C 0.0017

## Appendix A

**Table A1:** Differences in the median of leukocytes subpopulation percentages between healthy patients (**A**), patients with COVID-19 without interstitial lesions on chest X-ray ((**B**): COVID-19 X-ray (−)) and patients with COVID-19 with interstitial lesions on chest X-ray ((**C**): COVID-19 X-ray (+)). Data expressed as median (Q1–Q3).

[% of Total Leukocytes]	Control Group n = 20 (A) Median (Q1-Q3)	COVID-19 X-ray (-) n = 9 (B) Median (Q1-Q3)	COVID-19 X-ray (+) n = 14 (C) Median (Q1-Q3)	*p* < 0.05 * Group A-B-C ANOVA, Kruskal-Wallis	*p* < 0.05 * Group, in Groups Post-Hoc
Lymphocytes	38.3 (33.2–46.3)	49.8 (24.7–57.1)	27.6 (13.1–37.4)	0.0637	-
T Lymphocytes	30.5 (24.6–40.9)	36.2 (13.9–43.0)	19.4 (7.8–24.8)	* 0.0129	* A-C 0.0140
CD4 cells	18.6 (13.6–22.0)	23.1 (10.3–25.9)	8.0 (4.8–14.4)	* 0.0026	* A-C 0.0063 * B-C 0.0131
CD8 cells	10.5 (7.8–13.2)	9.4 (4.2–15.4)	8.5 (3.6–12.3)	0.4905	-
Ratio CD4/CD8	1.8 (1.5–2.2)	2.1 (1.4–3.5)	1.3 (0.6–2.4)	0.1460	-
Lymphocytes B [%]	3.9 (3.0–5.0)	2.7 (1.7–3.2)	2.1 (1.3–5.1)	0.0810	-
NK cells [%]	4.2 (2.8–7.0)	6.5 (4.1–9.1)	4.3 (1.5–9.1)	0.4628	-
Neutrophils	47.3 (42.1–57.3)	37.6 (29.7–69.4)	62.9 (49.4–77.5)	* 0.0229	* B-C 0.0230
Eosinophils [%]	2.2 (0.5–3.3)	1.7 (0.9–3.6)	0.9 (0.0–2.2)	* 0.0149	* A-C 0.0150
Basophils [%]	0.7 (0.5–1.2)	0.6 (0.3–1.5)	0.2 (0.0–0.5)	0.0511	-
Monocytes [%]	10.2 (6.8–11.5)	7.2 (4.1–8.1)	7.1 (6.2–11.9)	0.1611	-

## References

[1] Wang C., Horby P.W., Hayden F.G., Gao G.F. (2020). A novel coronavirus outbreak of global health concern. Lancet.

[2] Dong E., Du H., Gardner L. (2020). An interactive web-based dashboard to track COVID-19 in real time. Lancet Infect. Dis..

[3] Larici A.R., Cicchetti G., Marano R., Merlino B., Elia L., Calandriello L., Del Ciello A., Farchione A., Savino G., Infante A. (2020). Multimodality imaging of COVID-19 pneumonia: from diagnosis to follow-up. A comprehensive review. Eur. J. Radiol..

[4] Akl E.A., Blazic I., Yaacoub S., Frija G., Chou R., Appiah J.A., Fatehi M., Flor N., Hitti E., Jafri H. (2020). Use of Chest Imaging in the Diagnosis and Management of COVID-19: A WHO Rapid Advice Guide. Radiology.

[5] Lu R., Zhao X., Li J., Niu P., Yang B., Wu H., Wang W., Song H., Huang B., Zhu N. (2020). Genomic characterisation and epidemiology of 2019 novel coronavirus: implications for virus origins and receptor binding. Lancet.

[6] Shin H.S., Kim Y., Kim G., Lee J.Y., Jeong I., Joh J.S., Kim H., Chang E., Sim S.Y., Park J.S. (2019). Immune Responses to Middle East Respiratory Syndrome Coronavirus During the Acute and Convalescent Phases of Human Infection. Clin. Infect. Dis..

[7] Huang C., Wang Y., Li X., Ren L., Zhao J., Hu Y., Zhang L., Fan G., Xu J., Gu X. (2020). Clinical features of patients infected with 2019 novel coronavirus in Wuhan, China. Lancet.

[8] Quinti I., Lougaris V., Milito C., Cinetto F., Pecoraro A., Mezzaroma I., Mastroianni C.M., Turriziani O., Bondioni M.P., Filippini M. (2020). A possible role for B cells in COVID-19? Lesson from patients with agammaglobulinemia. J. Allergy Clin. Immunol..

[9] Li T., Qiu Z., Zhang L., Han Y., He W., Liu Z., Ma X., Fan H., Lu W., Xie J. (2004). Significant changes of peripheral T lymphocyte subsets in patients with severe acute respiratory syndrome. J. Infect. Dis..

[10] Wang F., Nie J., Wang H., Zhao Q., Xiong Y., Deng L., Song S., Ma Z., Mo P., Zhang Y. (2020). Characteristics of Peripheral Lymphocyte Subset Alteration in COVID-19 Pneumonia. J. Infect. Dis..

[11] Diao B., Wang C., Tan Y., Chen X., Liu Y., Ning L., Chen L., Li M., Liu Y., Wang G. (2020). Reduction and Functional Exhaustion of T Cells in Patients With Coronavirus Disease 2019 (COVID-19). Front. Immunol..

[12] Zheng M., Gao Y., Wang G., Song G., Liu S., Sun D., Xu Y., Tian Z. (2020). Functional exhaustion of antiviral lymphocytes in COVID-19 patients. Cell. Mol. Immunol..

[13] Xu Z., Shi L., Wang Y., Zhang J., Huang L., Zhang C., Liu S., Zhao P., Liu H., Zhu L. (2020). Pathological findings of COVID-19 associated with acute respiratory distress syndrome. Lancet Respir. Med..

[14] Moratto D., Chiarini M., Giustini V., Serana F., Magro P., Roccaro A.M., Imberti L., Castelli F., Notarangelo L.D., Quiros-Roldan E. (2020). Flow Cytometry Identifies Risk Factors and Dynamic Changes in Patients with COVID-19. J. Clin. Immunol..

[15] Odak I., Barros-Martins J., Bosnjak B., Stahl K., David S., Wiesner O., Busch M., Hoeper M.M., Pink I., Welte T. (2020). Reappearance of effector T cells is associated with recovery from COVID-19. EBioMedicine.

[16] Sims G.P., Ettinger R., Shirota Y., Yarboro C.H., Illei G.G., Lipsky P.E. (2005). Identification and characterization of circulating human transitional B cells. Blood.

[17] LeBien T.W., Tedder T.F. (2008). B lymphocytes: how they develop and function. Blood.

[18] Fink P.J. (2013). The biology of recent thymic emigrants. Annu. Rev. Immunol..

[19] van den Broek T., Borghans J.A.M., van Wijk F. (2018). The full spectrum of human naive T cells. Nat. Rev. Immunol..

[20] Boldt A., Borte S., Fricke S., Kentouche K., Emmrich F., Borte M., Kahlenberg F., Sack U. (2014). Eight-color immunophenotyping of T-, B-, and NK-cell subpopulations for characterization of chronic immunodeficiencies. Cytometry Part B Clin. Cytom..

[21] Martin M.D., Badovinac V.P. (2018). Defining Memory CD8 T Cell. Front. Immunol..

[22] Warnatz K., Schlesier M. (2008). Flowcytometric phenotyping of common variable immunodeficiency. Cytometry Part. B Clin. Cytom..

[23] Liu Z., Long W., Tu M., Chen S., Huang Y., Wang S., Zhou W., Chen D., Zhou L., Wang M. (2020). Lymphocyte subset (CD4+, CD8+) counts reflect the severity of infection and predict the clinical outcomes in patients with COVID-19. J. Infect..

[24] Sun H.B., Zhang Y.M., Huang L.G., Lai Q.N., Mo Q., Ye X.Z., Wang T., Zhu Z.Z., Lv X.L., Luo Y.J. (2020). The changes of the peripheral CD4+ lymphocytes and inflammatory cytokines in Patients with COVID-19. PLoS ONE.

[25] Zhang W., Li L., Liu J., Chen L., Zhou F., Jin T., Jiang L., Li X., Yang M., Wang H. (2020). The characteristics and predictive role of lymphocyte subsets in COVID-19 patients. Int. J. Infect. Dis. IJID Off. Publ. Int. Soc. Infect. Dis..

[26] Henry B., Cheruiyot I., Vikse J., Mutua V., Kipkorir V., Benoit J., Plebani M., Bragazzi N., Lippi G. (2020). Lymphopenia and neutrophilia at admission predicts severity and mortality in patients with COVID-19: A meta-analysis. Acta Bio-Med. Atenei Parm..

[27] Qun S., Wang Y., Chen J., Huang X., Guo H., Lu Z., Wang J., Zheng C., Ma Y., Zhu Y. (2020). Neutrophil-to-Lymphocyte Ratios Are Closely Associated with the Severity and Course of Non-mild COVID-19. Front. Immunol..

[28] Chen N., Zhou M., Dong X., Qu J., Gong F., Han Y., Qiu Y., Wang J., Liu Y., Wei Y. (2020). Epidemiological and clinical characteristics of 99 cases of 2019 novel coronavirus pneumonia in Wuhan, China: A descriptive study. Lancet.

[29] Wang D., Hu B., Hu C., Zhu F., Liu X., Zhang J., Wang B., Xiang H., Cheng Z., Xiong Y. (2020). Clinical Characteristics of 138 Hospitalized Patients With 2019 Novel Coronavirus-Infected Pneumonia in Wuhan, China. JAMA.

[30] Mathew D., Giles J.R., Baxter A.E., Oldridge D.A., Greenplate A.R., Wu J.E., Alanio C., Kuri-Cervantes L., Pampena M.B., D’Andrea K. (2020). Deep immune profiling of COVID-19 patients reveals distinct immunotypes with therapeutic implications. Science.

[31] De Biasi S., Lo Tartaro D., Meschiari M., Gibellini L., Bellinazzi C., Borella R., Fidanza L., Mattioli M., Paolini A., Gozzi L. (2020). Expansion of plasmablasts and loss of memory B cells in peripheral blood from COVID-19 patients with pneumonia. Eur. J. Immunol..

[32] Vaisman-Mentesh A., Gutierrez-Gonzalez M., DeKosky B.J., Wine Y. (2020). The Molecular Mechanisms That Underlie the Immune Biology of Anti-drug Antibody Formation Following Treatment With Monoclonal Antibodies. Front. Immunol..

[33] Peng Y., Mentzer A.J., Liu G., Yao X., Yin Z., Dong D., Dejnirattisai W., Rostron T., Supasa P., Liu C. (2020). Broad and strong memory CD4(+) and CD8(+) T cells induced by SARS-CoV-2 in UK convalescent individuals following COVID-19. Nat. Immunol..

[34] Gong F., Dai Y., Zheng T., Cheng L., Zhao D., Wang H., Liu M., Pei H., Jin T., Yu D. (2020). Peripheral CD4+ T cell subsets and antibody response in COVID-19 convalescent individuals. J. Clin. Investig..

[35] Sekine T., Perez-Potti A., Rivera-Ballesteros O., Stralin K., Gorin J.B., Olsson A., Llewellyn-Lacey S., Kamal H., Bogdanovic G., Muschiol S. (2020). Robust T Cell Immunity in Convalescent Individuals with Asymptomatic or Mild COVID-19. Cell.

[36] Westmeier J., Paniskaki K., Karakose Z., Werner T., Sutter K., Dolff S., Overbeck M., Limmer A., Liu J., Zheng X. (2020). Impaired Cytotoxic CD8(+) T Cell Response in Elderly COVID-19 Patients. mBio.

[37] Elsaesser H.J., Mohtashami M., Osokine I., Snell L.M., Cunningham C.R., Boukhaled G.M., McGavern D.B., Zuniga-Pflucker J.C., Brooks D.G. (2020). Chronic virus infection drives CD8 T cell-mediated thymic destruction and impaired negative selection. Proc. Natl. Acad. Sci. USA.

